# Transcriptional regulation of FOXP3 requires integrated activation of both promoter and CNS regions in tumor-induced CD8^+^ Treg cells

**DOI:** 10.1038/s41598-017-01788-z

**Published:** 2017-05-09

**Authors:** Sreeparna Chakraborty, Abir K. Panda, Sayantan Bose, Dia Roy, Kirti Kajal, Deblina Guha, Gaurisankar Sa

**Affiliations:** Division of Molecular Medicine, Bose Institute, P-1/12, CIT Scheme VII M, Kolkata, 700054 India

## Abstract

T-regulatory cells are an upsurge in the tumor microenvironment and induce immune-evasion. CD4^+^ Treg cells are well characterized whereas the role of CD8^+^ Tregs in cancer has recently started to crease attention. Here, we report an augmentation CD8^+^FOXP3^+^ Tregs in breast tumor microenvironment. FOXP3, the lineage-specific transcription factor, is a dominant regulator of Treg cell development and function. FOXP3 is induced preferentially by divergent signaling in CD4^+^ Treg cells. But how *FOXP3* is induced and maintained in tumor-CD8^+^ Tregs is the Cinderella of the investigation. We observed that RUNX3, a CD8^+^ lineage-specific transcription factor, binds at the *FOXP3*-promoter to induce its transcription. In addition to promoter activation, involvement of *cis*-elements CNS1 and CNS2 in the transcriptional regulation of *FOXP3* was also evident in these cells. SMAD3 binds to CNS1 region and acts as transcription inducer, whereas GATA3 plays a temporal role in the *FOXP3* transcription by differential chromatin modification in CNS regions. In CNS1 region, GATA3 acts as a repressor for *FOXP3* in naïve CD8^+^ T cells. Whereas in CD8^+^ Tregs, GATA3 binds directly at CNS2 region and persuaded the maintenance of *FOXP3*. Therefore, the intervention of these concerted transcriptional machinery may have a therapeutic potential in immunotherapy of cancer.

## Introduction

T-regulatory cells (Tregs) play a pivotal role in the development and maintenance of peripheral immune tolerance as defects in their compartment lead to severe autoimmune diseases. But these functions also have some detrimental effects on tumor immunosurveillance and antitumor immunity. The association of FOXP3-positive CD4^+^ Treg cells with prognosis and progression of cancer is well established^1, 2^, but the role of CD8^+^ Tregs in cancer is not clearly deciphered yet, due to lack of universal phenotypic signature which could distinguish CD8^+^ Tregs from conventional CD8^+^ T cells.

Prevalence of distinct subset of CD8^+^ Treg cells are reported in colon cancer (CD8^+^CD25^+^FOXP3^+^ cells), prostate cancer (CD25^+^CD122^+^FOXP3^+^ and partly GITR^+^ cells), non-small cell lung cancer (CD8^+^CD28^−^ cells) and in ovarian cancer (CD8^+^CCR7^+^IL10^+^ cells)^3–6^. Separate subsets of CD8^+^ Tregs use different mechanisms of suppression^7^, but the immunosuppressive properties which ensure tumor immune escape were commonly defined on their ability to secret IL10 or TGFβ. FOXP3, a member of forkhead/winged-helix family, is a lineage-specific master regulator of Treg cells which hijacks the transcription machinery for effector T cells and functionally converts them into Treg cells^8^. The role of a different transcription factor in the epigenetic regulation of FOXP3 in CD4^+^ Treg is already considered well. Though, CD8^+^ Treg cells were identified in the 1970s by Grehson *et al*.^9^, a precise molecular mechanism that regulates the *FOXP3* expression in CD8^+^ Treg subset is yet to be explored.

In CD4^+^ Treg cells, FOXP3 induction is coupled with the activation of TCR/NFAT-NFκB-signalling or by TGFβ/SMAD3-signaling^2, 10^. In addition to these factors other, transcription factors, *viz*; GATA3, RUNX3, and RUNX1 also play pivotal roles in the regulation of *Foxp3* gene expression^11, 12^. FOXP3 is already well studied as a transcriptional activator as well as a repressor. FOXP3 up-regulates the expression of Treg cell-associated molecules including CD25 and CTLA4^13, 14^ and also induce the IL10 production by co-transcriptional regulation along with STAT3^1^. On the other hand, FOXP3 transcriptionally represses expression of the cytokine IL2 in Treg cells^15^. So, to understand immune tolerance and immune homeostasis by CD8^+^ Treg cell, how the FOXP3 expression is transcriptionally controlled in CD8^+^ Treg, is a critical question yet to be fully addressed.

Here, we report the prevalence of CD8^+^ Treg cells in breast tumor microenvironment which expresses high-level of FOXP3. Since CTLA4 is transcriptionally activated by FOXP3, we used this co-stimulatory molecule as a surface signature for the isolation of tumor-CD8^+^ Treg cells for our study. Exploiting these cells we could successfully show that transcriptional activation of FOXP3-promoter is associated with chromatin modification and binding of SMAD3, GATA3, and RUNX3 at the different non-coding conserved sequence (CNS) and the promoter regions of FOXP3. This study may open a new way to target CD8^+^ Treg cells and thus potentiates the antitumor immunity during cancer immunotherapy.

## Results

### Prevalence of CD8^+^CD25^+^FOXP3^+^ Treg cells in breast tumor milieu

**Figure 1:**
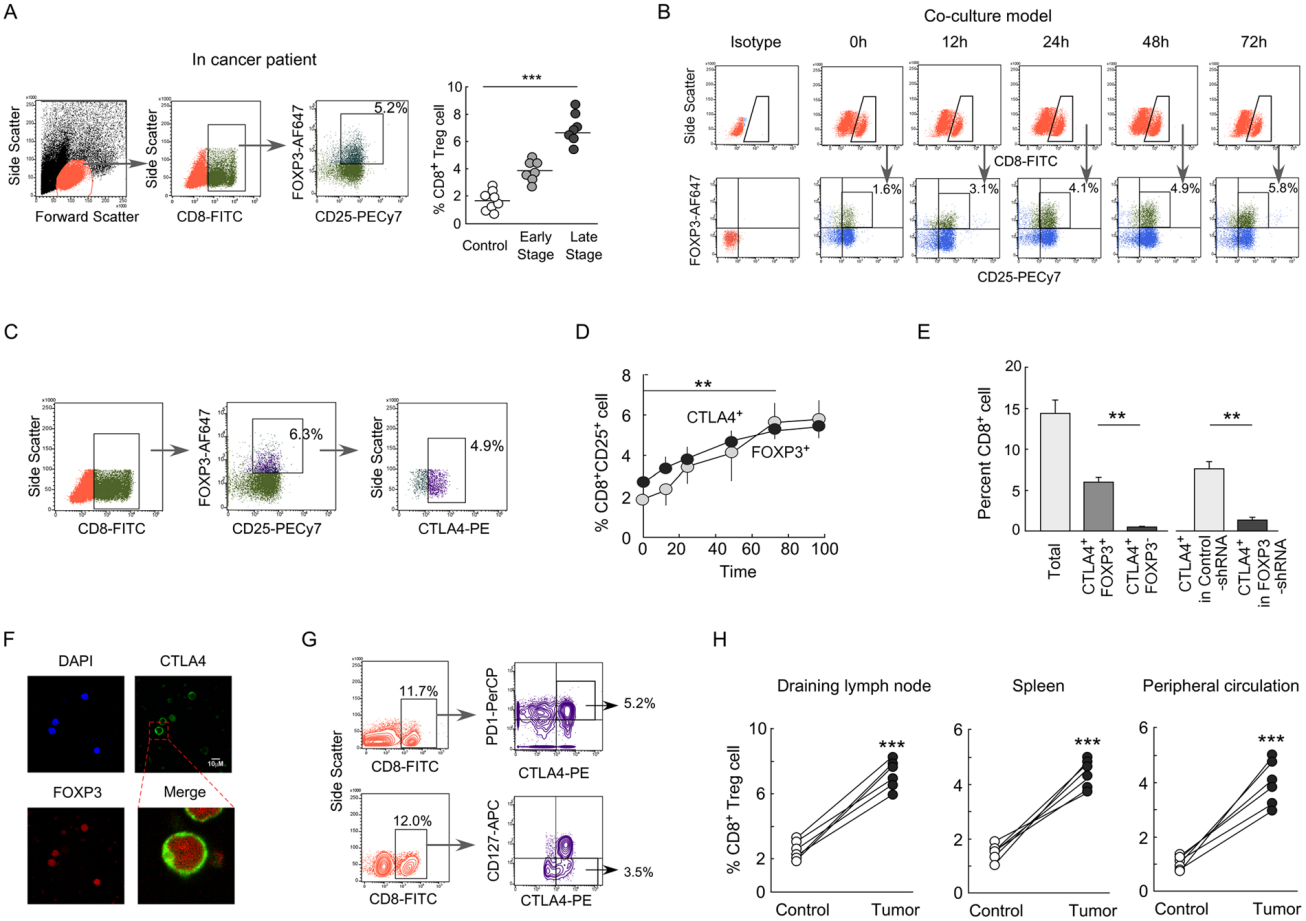
Prevalence of CD8^+^CD25^+^FOXP3^+^ Treg cell in breast tumor-microenvironment. (**A**) T Lymphocytes were isolated from peripheral circulations of breast cancer patients and were subjected to CD8/CD25/FOXP3 staining. Lymphocytes population was first gated to study the percentage of CD8^+^CD25^+^ cells which was further gated to observe percentage of CD25^+^FOXP3^+^ cells within it. Numbers in the box indicate respective percent cells (*left panel*). Percentage CD8^+^CD25^+^FOXP3^+^ T cells from different stages of breast cancer patients were depicted graphically (*right panel*). Data are representative as the mean ± SEM and are the cumulative results from twelve female breast cancer (early stage, n = 6; late stage, n = 6) patients and six healthy age/sex-matched individuals. (**B**) In our *in vitro-*generated tumor microenvironment, CD8^+^CD25^+^ cells were analyzed for their FOXP3-positivity at various time of co-culture with primary breast tumor cells. (**C**) CD8^+^CD25^+^ cells were gated to study their FOXP3-positivity, and the FOXP3^+^ cells were further assayed for their CTLA4-positivity. (**D**) *In vitro* developmental profiles of CD8^+^CD25^+^CTLA4^+^ and CD8^+^CD25^+^FOXP3^+^ T cells. (**E**) The graphical representation showed the percentages of CD8^+^/CD8^+^CTLA4^+^FOXP3^−^ (exhausted T cell), CD8^+^CTLA4^+^FOXP3^+^ (CD8^+^ Treg) cells and CD8^+^CTLA4^+^ cells generated in FOXP3-shRNA transduced conditions. (**F**) The confocal microscopic data showed the CTLA4 and FOXP3-positivity in CD8^+^ T cell cultured in *in vitro* tumor microenvironment. (**G**) Flow cytometric representation showed CD8^+^CTLA4^+^PD1^+^ and CD8^+^CTLA4^+^CD127^−^ cell populations within total lymphocytes developed in *in vitro-*generated tumor model and highlighted by outlined box. (**H**) The percentages of CD8^+^CD25^+^Foxp3^+^ Treg cells in different immune compartments of normal and breast tumor-bearing mice were represented graphically. Isotype-matched control antibodies were used for all flow cytometric experiments. Data are representative as the mean ±SEM and are the cumulative results of five independent experiments. *p < 0.05, **p < 0.01, ***p < 0.001.

Till date, CD4^+^ Treg is known to be an important contributor in tumor development and maintenance^16^, but the role of CD8^+^ Treg in the tumor microenvironment is yet to be explored. CD8^+^ Treg that reported to be present in prostate or colon cancer cancers is FOXP3-positive^3, 4^. Similarly, we observed the prevalence of CD8^+^CD25^+^FOXP3^+^ T cells in the peripheral circulation of patients with advanced breast cancer, in comparison to age-sex matched healthy donors (Fig. 1A). For better elucidation of human CD8^+^ Treg cells features and their functions in the tumor microenvironment, we developed an *in vitro* co-culture model in which isolated human lymphocytes were layered over the bed of monolayers of cells, obtained from a primary breast tumor. Then, to identify the developmental stages of CD8^+^ Treg cells in breast tumor microenvironment, we monitored the CD25- and FOXP3-positivity within CD8^+^ T cells. It was observed that there was a significant increase in CD8^+^CD25^+^FOXP3^+^ T cell population with time (Fig. 1B) indicating that we could successfully mimic the tumor microenvironment in *in vitro* condition.

Report by Wang *et al*. showed that transient FOXP3 induction occurs in activated T cells^17^. To nullify our *in vitro*-generated CD8^+^ Treg cells as transiently-FOXP3-expressing activated CD8^+^ T cells, we incubated both the activated (CD3/CD28/IL2 stimulated) CD8^+^ T cells and *in vitro*-generated CD8^+^ Treg cells in fresh media for 6 days and the status of FOXP3 was checked. Unlike activated CD8^+^ T cells, *in vitro*-generated CD8^+^ Treg cells showed a sustained expression of FOXP3 indicating that these cells are true CD8^+^ Treg cells (Supplementary Fig. S1A). In addition, unlike activated CD8^+^ T cells, these CD8^+^ Treg cells produce a low level of IL2 (Supplementary Fig. S1B), as because FOXP3 represses the IL2 transcription^18^. All these information suggest that the FOXP3-expressing CD8^+^ T cells generated in *in vitro* tumor microenvironment are not activated CD8^+^ T cells. Interestingly, when CD8^+^ T cells were co-cultured with normal kidney epithelial (NKE) cells, no CD8^+^CD25^+^FOXP3^+^ population were induced indicating that the induced CD8^+^ Treg cells are tumor specific and just any anonymous response to different cell lineages (Supplementary Fig. S1C).

However, being a nuclear protein, FOXP3 would have restricted value in the isolation of CD8^+^ Treg cells for mechanistic study. No specific surface marker of CD8^+^ Treg has been yet established. Therefore, we next decided to identify a specific surface marker for tumor-CD8^+^ Treg cells, so that we can isolate those cells for further studies. CTLA4, an FOXP3-transactivated co-stimulatory molecule is now considered as a surface signature of CD4^+^ Treg cells, so we checked the CTLA4-positivity within CD8^+^CD25^+^ T cells and observed that almost all FOXP3-positive are cells CTLA4-positive (Fig. 1C). Aiding to this, we observed that CD8^+^CD25^+^CTLA4^+^ T cell population shows a coherent pattern with CD8^+^CD25^+^FOXP3^+^ T cells (Fig. 1D). Recent reports suggest that in tumor microenvironment T cells terminally differentiate into ‘exhausted’ T-cells which are CTLA4-/PD1-positive^19, 20^. So, before considering CTLA4 as a marker of CD8^+^FOXP3^+^ Treg cells, we checked the presence of total CD8^+^ cells, CD8^+^CTLA4^+^FOXP3^−^ exhausted T cells and CD8^+^CD25^+^CTLA4^+^FOXP3^+^ Treg cells in our experimental condition. We observed that in our *in vitro* generated tumor-microenvironment, CTLA4^+^ ‘exhausted’ T cells percentage were very low (<1%) as compared to FOXP3^+^CTLA4^+^CD8^+^ Treg cells (6.5%) (Fig. 1E) and this indicated that isolated CD8^+^CD25^+^CTLA4^+^ T cell population predominantly comprised of CD8^+^ Treg cells. For further confirmation, we have co-cultured FOXP3-ablated CD8^+^ T cells in *in vitro* tumor milieu and checked the status of CTLA4-positivity. Our flow cytometric data suggest that FOXP3 knockdown suppressed CTLA4 induction in these cells indicating that nearly all CD8^+^CD25^+^CTLA4^+^ cells are essentially CD8^+^ Treg cells (Fig. 1E). Our confocal microscopic data further confirmed that the CD8^+^FOXP3^+^ cells are invariably CTLA4-positivein in nature (Fig. 1F).

We also checked the CD127-negativity and PD1-positivity, the other surface markers of Tregs^21, 22^, within these cells (Fig. 1G) which also confirmed their Treg phenotype. However, for more precise confirmation of these Treg cells as bona fide Tregs, we compared the phenotypes of this CD8^+^ Treg cells generated in *in vitro* tumor-microenvironment with that present in the peripheral circulation of breast cancer patients. It was also observed that the *in vitro*-generated Treg showed coherent phenotype with that of the patients-derived Treg cells (Supplementary Fig. S1D–F). All these results together suggested that the *in vitro* tumor microenvironment-generated CD8^+^ Treg cells are true Treg population similar to that present in tumor milieu.

To study the distribution of CD8^+^ Treg cell population in the different immune compartment, we developed breast tumor model by implanting isogenic 4T1 (breast cancer) cells in the mammary-fat pad of BALB/c mice. After 21 days, those mice were sacrificed, and CD8^+^CD25^+^Foxp3^+^ Treg cells population were monitored in different immune compartments. As expected it was observed that CD8^+^ Treg populations were augmented in all the popliteal lymph nodes, spleen and circulatory compartments of those tumor-bearing mice (Fig. 1H).

### Tumor-induced CD8^+^ Treg cells are immunosuppressive in nature

**Figure 2:**
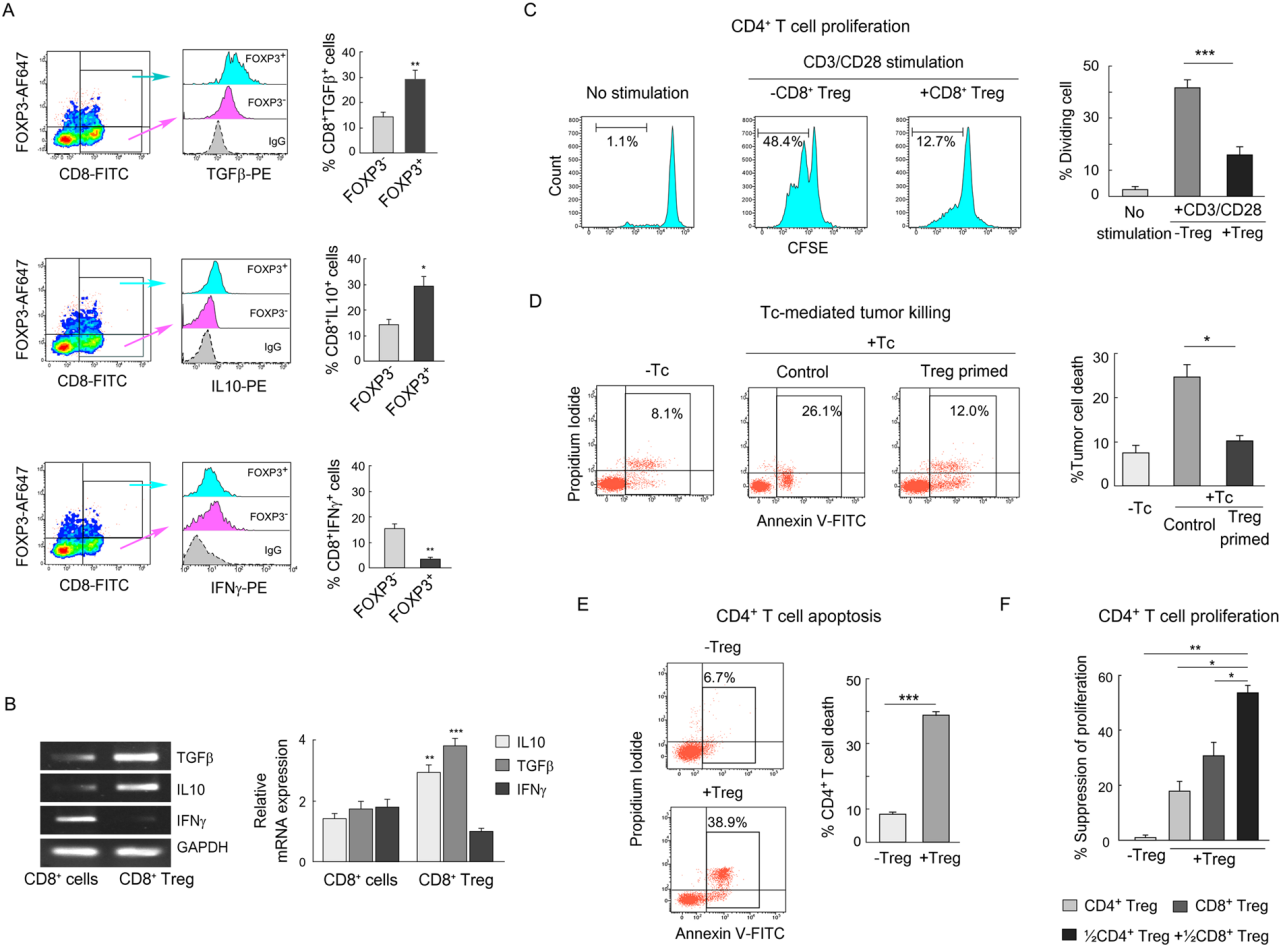
Immunosuppressive properties of tumor-induced CD8^+^ Treg cells. (**A**) Flow cytometric histoplot represented TGFβ (*first panel*), IL10 (*second panel*), and IFNγ (*third panel*) positivity within CD8^+^FOXP3^+^ Treg cells (*blue*) and CD8^+^FOXP3^−^ cells (*pink*). Percentages of the respective population were represented graphically in *right panels*. (**B**) The relative transcript levels of TGFβ, IL10, and IFNγ were analyzed by RT-PCR (*upper panel*) and quantified by qPCR (*lower panel*). The gel bands were cropped, whole gel pictures were given in Supplementary Fig. S2A. (**C**) CD4^+^ responder T cell proliferation was measured by CFSE-dilution assay in presence and absence of isolated tumor-CD8^+^ Treg cells (CD8^+^CD25^+^CTLA4^+^ T cells). Responder cells were stimulated with anti-CD3/-CD28 antibodies and rIL2 for 96 h. Numbers in outlined areas indicated the percent of dividing cells in each histo-plot and presented statistically (*right panel*). (**D**) CD8^+^ cytotoxic cells (Tc) were primed with CD8^+^ Treg cells. Control and Treg-primed Tcs were then co-incubated separately with breast tumor cells for a 48 h and apoptotic index of the tumor cells i.e. CD8-negative population, were analyzed flow cytometrically by Annexin-V/PI-positivity and represented statistically (*right panel*). (**E**) CD4^+^ T cell apoptosis was determined in presence and absence of tumor-CD8^+^ Treg cells and analyzed statistically. (**F**) Percent suppression of CD4^+^ responder cells was determined by CFSE dilution assay in presence or absence of CD4^+^ and CD8^+^ Treg cells alone or a combination. Isotype-matched control antibodies were used for all flow cytometric experiments. Data are representative as the mean ± SEM and are the cumulative results of five independent experiments. *p < 0.05, **p < 0.01, ***p < 0.001. *GAPDH* was used as internal loading control in RT-PCR and qPCR.

It is well acknowledged that Treg cells mediate their suppressive function through the secretion of different immunosuppressive cytokines, suppression of effector T cell expansion and inhibition of Tc-mediated cytotoxicity^23, 24^. Therefore, we next sought to determine the cytokine profile of these FOXP3^+^CD8^+^ Treg cells generated in *in vitro* tumor model. Results showed that FOXP3^+^CD8^+^ T cells produce high-level of IL10 and TGFβ (Fig. 2A, *first and second panel*) as compared to their FOXP3-negative counterpart. Whereas, in the case of IFNγ, we observed that CD8^+^FOXP3^+^ cells produce the very low level of IFNγ in comparison to CD8^+^FOXP3^−^ control cells (Fig. 2A, *third panel*). The transcript level of IL10, TGFβ and IFNγ cytokine showed a coherent pattern with their protein level (Fig. 2B).

To study Treg-mediated suppression of T-responder cells in detail, we co-cultured CFSE loaded responder (CD4^+^) cells with isolated CD8^+^ Treg cells and with CD8^+^ T cells as a control for 48 h. It was evident from CFSE-dilution assay that (Fig. 2C) CD8^+^ Treg significantly suppressed responder cells proliferation. We were interested whether this CD8^+^ Treg cells can also inhibit effecter CD8^+^ T cell (Tc) cell functions. Therefore, we used another co-culture system in which we first primed Tc cells with CD8^+^ Treg cells for 48 h and after that CD8^+^CTLA4^−^ cells (Tc) were isolated from that co-culture. Next, tumor cells (MCF7) were co-cultured with this Treg-primed Tc cells and with untreated Tc as a control. After the co-culture period, Tc-mediated tumor cell killing was determined by Annexin V/PI-positivity (Fig. 2D) and the results showed that cytotoxic activity of Treg-primed Tc cells was significantly hindered as compared to control Tc cells. These CD8^+^ Treg cells could induce the death of CD4^+^ T cell which is also a hallmark of their suppressive property (Fig. 2E) similar to the CD8^+^ Treg cells isolated from tumor patients (Supplementary Fig. S2B–D). It was observed that the suppressive activities were significantly increased with the increased proportions of CD8^+^ Treg cells and there was no significant difference in the suppressive activities between *in vitro*-generated Treg cells and the patient-derived Treg cells (Supplementary Fig. S2B–D).

Now, in this scenario, it was noticeable that in tumor milieu, both CD4^+^ and CD8^+^ Treg cells are equipped with immunosuppressive properties which compelled us to check whether both of these T-regulatory cells work together to ensure tumor immune escape. To test the possibility, we again loaded CD4^+^ responder cells with CFSE and then co-culture separately with CD4^+^ Treg, CD8^+^ Tregs alone or in combination. It was observed that both the Tregs suppressed proliferation of CD4^+^ responder cells and interestingly when these responder cells were co-incubated with both CD4^+^ and CD8^+^ Tregs in combinations, these cells suppressed those CD4^+^ T cell proliferation synergistically (Fig. 2E).

### RUNX3 enhanced the promoter activity of FOXP3 in tumor-induced CD8^+^ Treg cells

**Figure 3:**
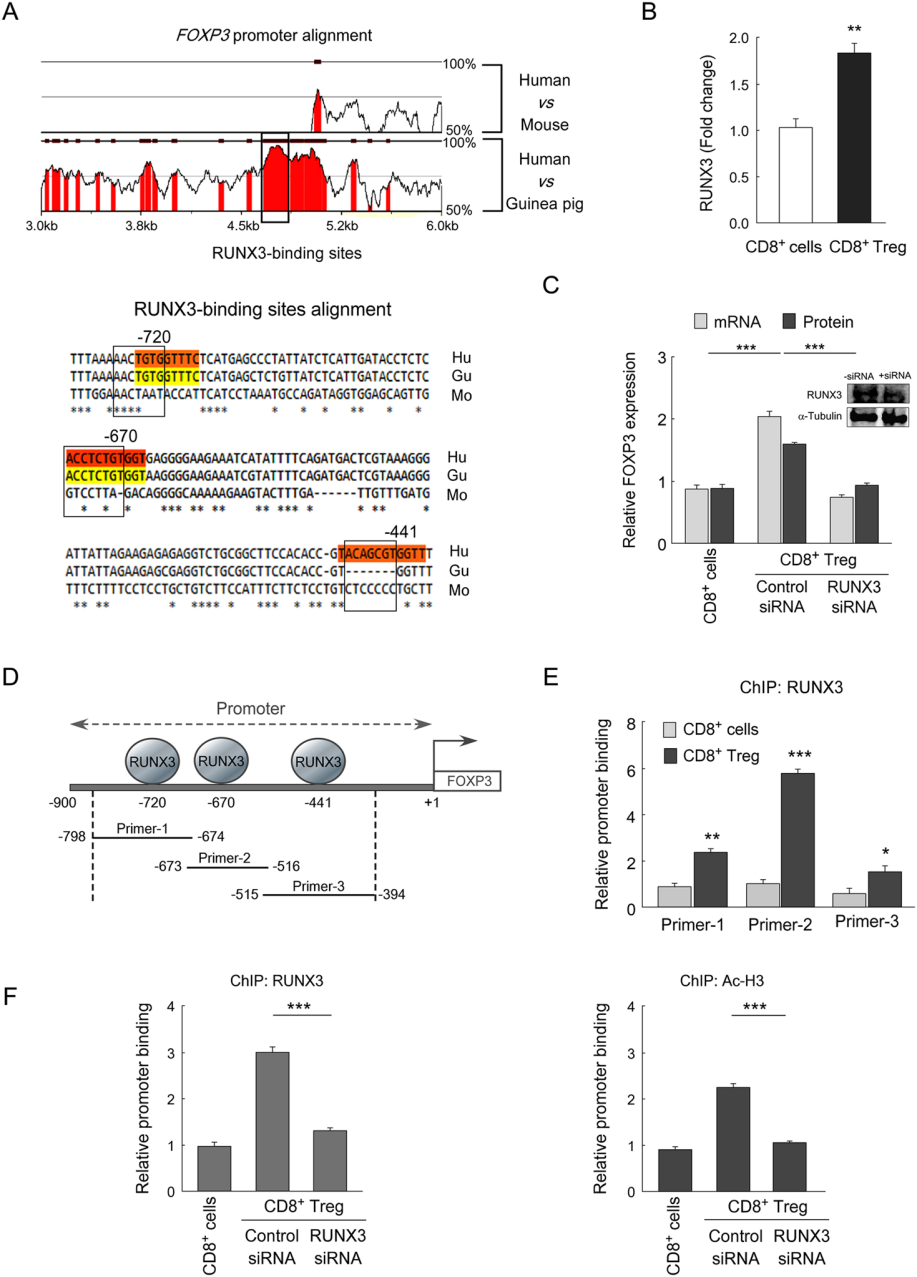
RUNX3 enhances promoter activity of FOXP3 in CD8^+^ Treg cells. (**A**) Multiple sequence alignment between human FOXP3, mouse Foxp3 and guinea-pig FoxP3 genome (human accession NC_000023.11; mouse accession NC_000086.7; guinea pig accession NT_176287.1) depicted the RUNX3-binding sites on *FOXP3*-promoter, and the region is conserved between human and guinea-pig (*upper panel*). rVista criteria that were applied require 90% similarity for at least 35 bp length. In the bottom, CLUSTALW alignment of human/mouse/guinea-pig *FOXP3* genome depicted three conserved RUNX3-binding sequences at promoter region. The outlined area indicated RUNX3-binding sites. (**B**) The relative level of RUNX3 in control CD8^+^ cells and tumor-CD8^+^FOXP3^+^ Treg cells was determined by mean fluorescence intensity value in flow cytometry. (**C**) Relative *FOXP3*-mRNA and protein expressions in control CD8^+^ T cells and control-siRNA/RUNX3-siRNA transfected tumor-CD8^+^ Treg cells were analyzed by qPCR and flow cytometry respectively. Transfection efficiency was determined by Western blot (*inset*). (**D**) Schematic diagram representing different RUNX3-binding regions on *FOXP3*-promoter and the sequential order of the overlapping primer sets (primer-1: −798 bp to −674 bp; primer-2: −673 bp to −516 bp; primer-3: −515 bp to −394 bp) to analyze RUNX3 binding on *FOXP3*-promoter by ChIP assay. (**E**) Quantitative-PCR data depicted relative binding of RUNX3 on those three putative responsive elements in the *FOPX3* promoter. (**F**) In control CD8^+^ T cells and control-siRNA/RUNX3-siRNA transfected tumor-CD8^+^ Treg cells, the relative binding of RUNX3 (*left panel*) and Ac-H3 (*right panel*) at −670 bp responsive element (primer-2) were analyzed by ChIP assay. Data are representative as the mean ± SEM and are the cumulative results of five independent experiments. *p < 0.05, **p < 0.01, ***p < 0.001.

Considering a central role of FOXP3 in Treg cell differentiation and function, its transcriptional regulation in CD8^+^ Treg cells is crucial to be understood. It is well acknowledged that runt domain transcription factor, RUNX3, plays a critical role in the lineage determination of CD8^+^ T cells from CD4/CD8-positive progenitor cells^25^. And our *in silico*, human *FOXP3*-promoter analysis predicted three putative RUNX3-binding sites at −720 bp, −670 bp and −441 bp upstream of the transcription start site which is conserved between human and guinea pig (Fig. 3A). All these information tempted us to check whether RUNX3 has any role in the transcriptional activation of *FOXP3* in tumor-induced CD8^+^ Treg cells or not. It was observed that level of RUNX3 was significantly high in these Treg cells as compared to their normal counterpart (Fig. 3B). To study the involvement of RUNX3 in *FOXP3* transcription we transfected CD8^+^ T cells with control- or RUNX3-siRNA and then cultured these cells in *in vitro* tumor microenvironment. We observed that CD8^+^FOXP3^+^ Treg cells generation was significantly perturbed and *FOXP3-*mRNA expression was found to be very low in RUNX3-ablated CD8^+^ Treg cells as compared to control-siRNA transfected cells (Fig. 3C). This information indicated a pivotal role of RUNX3 in FOXP3 regulation in tumor-CD8^+^ Treg cells. Next, we checked its direct binding to the *FOXP3* promoter. For the same, we designed three sets of overlapping primers from −798 bp to −394 bp region of the *FOXP3* promoter as determined by our *in silico* analysis (Fig. 3D). All these three RUNX3-binding sites showed positive ChIP-signals in CD8^+^ Treg cells, and amongst them relative binding of RUNX3 was maximum at the −670 bp site, and this site was found to be hyper-acetylated (Ac-H3) in CD8^+^ Treg cells in comparison to control CD8^+^ T cells (Fig. 3E). To further validate our hypothesis, we transfected CD8^+^ T cells with control-/RUNX3-siRNA before co-culturing in tumor milieu and then did ChIP assay after 72 h of co-culture. Results showed that ChIP signal for both RUNX3 and Ac-H3 were decreased significantly in RUNX3-ablated condition (Fig. 3F). Together all these results suggested that RUNX3 play a vital role in FOXP3 transcription in tumor-CD8^+^ Treg by enhancing the promoter activity.

### SMAD3 induces FOXP3 transcription in CD8^+^ Treg cells by docking at CNS1 region

**Figure 4:**
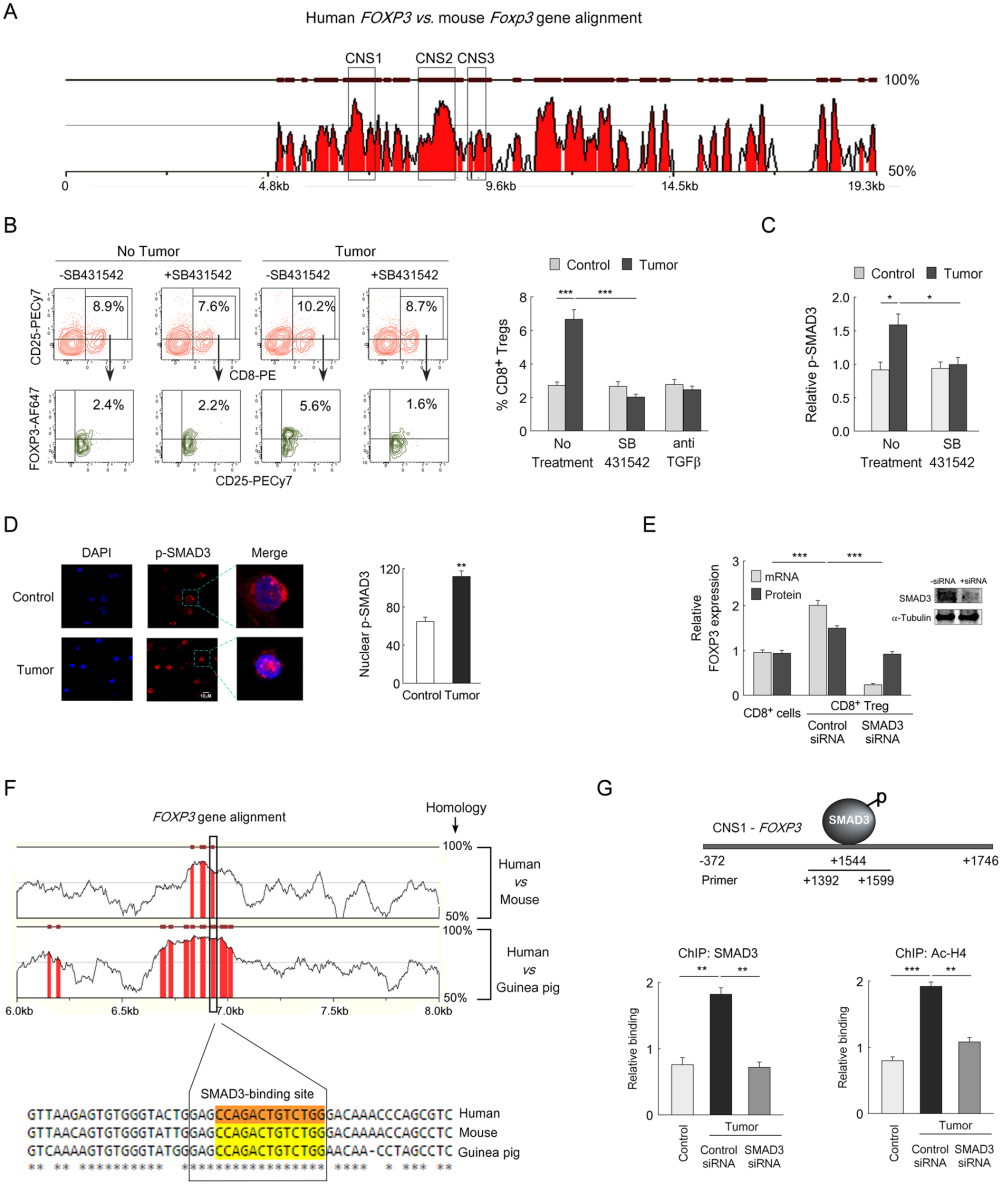
SMAD3 mediated transcriptional regulation of FOXP3 in CD8^+^ Treg cells. (**A**) Multiple sequence alignment between human *FOXP3* and mouse *Foxp3* depicted the non-coding conserved sequence; *viz*. CNS1, CNS2, CNS3. Criteria that were applied for rVista analysis required 80% identity for at least 100-bp length. (**B**) Lymphocytes were isolated from a healthy individual and were cultured in media alone or in *in vitro*-generated tumor milieu in presence or absence of TGFβ receptor inhibitor SB431542 and a percent population of CD8^+^CD25^+^FOXP3^+^ Treg cells were analyzed by flow cytometry (*left panel*). The graphical representation depicted percent CD8^+^CD25^+^FOXP3^+^ Treg cells generated in SB431542-treated/TGFβ-neutralizing antibody-treated conditions (*right panel*). (**C**) Graphical plot represents the relative changes in phospho-SMAD3 levels in the SB431542-treated control CD8^+^ cell as well as CD8^+^FOXP3^+^ Treg cells. (**D**) Representative confocal images depicted nuclear translocation of p-SMAD3 in control CD8^+^ cells and tumor-CD8^+^ Treg cells (magnification 60x; scale bar: 5 μM) (*left panel*). Nuclear translocation of p-SMAD3 was analyzed by ImageJ software and depicted graphically (*right panel*). (**E**) The relative change in FOXP3 expression at transcript and protein level was determined in control CD8^+^ cell and SMAD3-siRNA-transfected CD8^+^ Treg cells by qPCR and flow cytometry respectively. Transfection efficiency was determined by Western blot (*inset*). (**F**) rVista alignment of human/mouse/guinea-pig *FOXP3* genome sequences. Criteria required 90% similarity for at least 35 bp length. The conserved regions were in the red. The outlined area indicated SMAD3-binding sites in CNS1 enhancer region (*above*). Sequence conservation of the SMAD3-binding site in CNS1 region of *FOXP3* genome between human/mouse/guinea-pig was analyzed by CLUSTALW (*bottom*). The highlighted area indicated SMAD3-binding sequence. (**G**) Schematic representation of SMAD3-binding sites at CNS1 region and the used primer set: +1392 bp to +1599 bp (*above*). In the bottom, qPCR data showed relative SMAD3 binding (*left panel*) and histone-H4 acetylation (*right panel*) in control CD8^+^ T cells and control-/SMAD3-siRNA transfected tumor-CD8^+^ Treg cell. Data are representative as the mean ±SEM and are the cumulative results of five independent experiments. *p < 0.05, **p < 0.01, ***p < 0.001.

In addition to the promoter region three conserved noncoding sequences (CNSs) in the *FOXP3* locus, *viz*. CNS1, CNS2 and CNS3^26, 27^, also regulate and maintain *FOXP3* transcription (Fig. 4A). Among them CNS1 is known to contain the TGFβ-responsive element, CNS2 is important in maintaining *FOXP3* expression in thymus-derived nTreg cells, and CNS3 contains a highly conserved CpG-rich island which is known to exhibit enhancer activity in response to TCR stimulation only^10, 28^. In our previous work, we have observed that breast tumor cells secrete high-level of TGFβ^2^. To test whether TGFβ-signalling plays any role in *FOXP3* induction in tumor-CD8^+^ Treg cells, we used specific pharmacological TGFβ receptor inhibitor (SB431542) or TGFβ neutralizing antibody. In this, *in vitro*-generated tumor microenvironment, we observed that perturbation of TGFβ-signalling significantly inhibited FOXP3^+^CD8^+^ Treg cell generation (Fig. 4B). TGFβ-mediated external stimuli must be transmitted to the nucleus through SMADs to regulate FOXP3 expression in CD8^+^ Treg cells. Among different isoforms of SMAD protein, SMAD3 is known to play a key role in FOXP3 induction in CD4^+^ Treg cells^2^. Therefore, to test events further downstream in the TGFβ pathway, we observed that augmentation of phospho-SMAD3, as well as their nuclear translocation, was enhanced in tumor-CD8^+^ Treg in comparison with control CD8^+^ T cells (Fig. 4C and D). And SMAD3-ablation significantly blocked the expression of FOXP3 both transcriptional and translational levels (Fig. 4E).

Since FOXP3-CNS1 region is crucial for TGFβ-mediated FOXP3 induction, we next asked the question whether in tumor-CD8^+^ Treg cells SMAD3 regulates CNS1 activity or not. Our *in silico* analysis showed that CNS1 sequence is almost 70% conserved between human, mouse and guinea pig (Fig. 4A). Within this CNS1 region, we have identified an SMAD3-binding site which is 90% conserved between human, mouse and guinea pig as determined by multiple sequence alignment (Fig. 4F). Bioinformatics analysis showed that SMAD3-binding site is at +1544 bp, within the CNS1 region which is extended from −372 bp upstream of the transcription start site to +1746 bp (Fig. 4G, *upper panel*). This SMAD3-binding sites showed a positive ChIP-signals in at the responsive element in CNS1 region of *FOXP3* genome (Fig. 4G, *bottom left panel*), and this site was found to be hyper-acetylated (Ac-H4) in CD8^+^ Treg cells in comparison to control CD8^+^ T cells (Fig. 4G, *bottom right panel*). SMAD3-ablation completely abrogated such positive ChIP-signals for both the SMAD3 and the AcH4 (Fig. 4, *bottom panels*). All these data indicated an SMAD3-mediated transcriptional activation of *FOXP3* by active chromatin modification.

### GATA3 plays a dual role in transcriptional regulation of FOXP3 in CD8^+^ Treg cells

**Figure 5:**
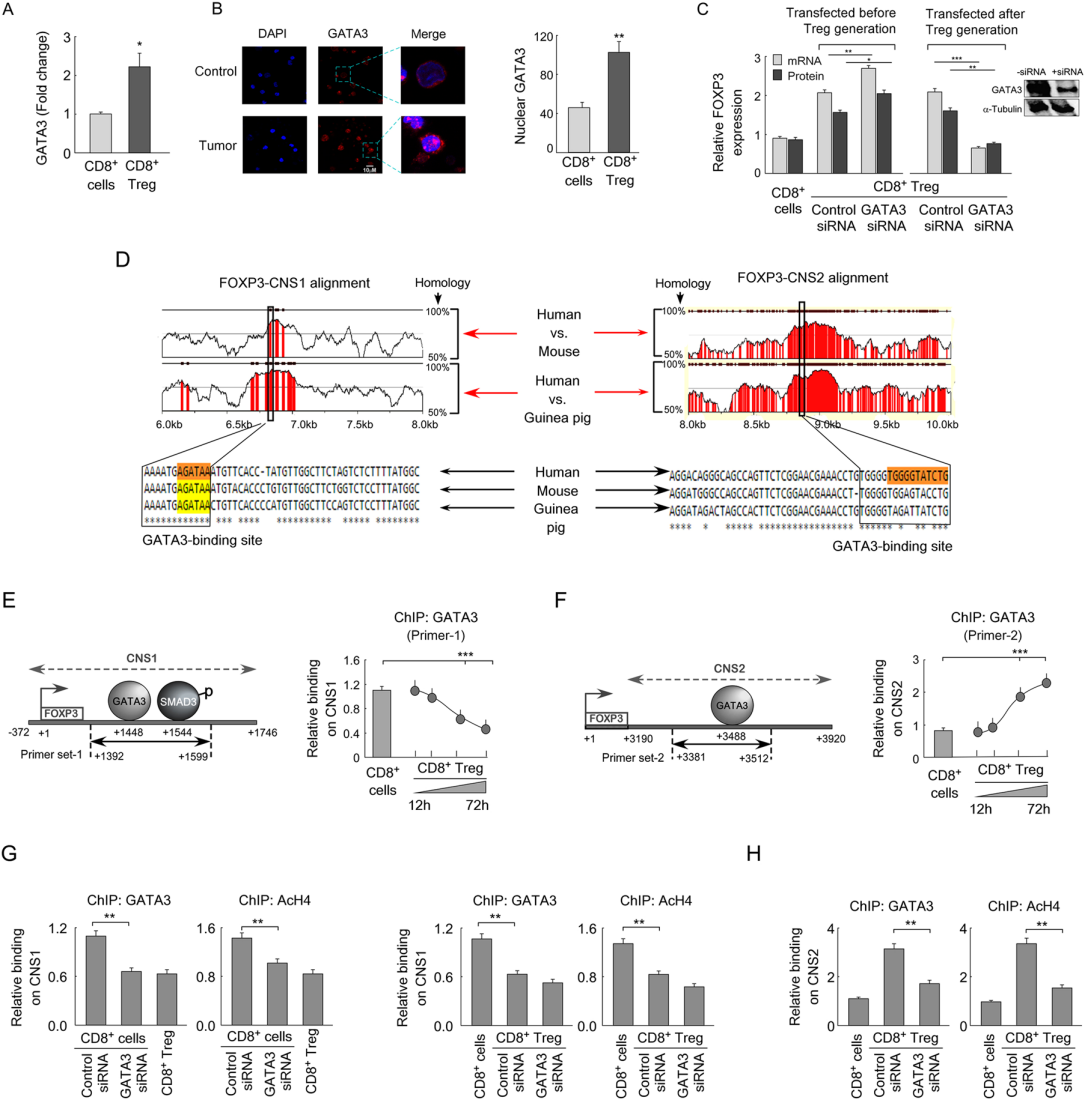
GATA3 play a dual role in FOXP3 regulation in CD8^+^ Treg cells. (**A**) The graphical diagram represents relative levels of GATA3 in control CD8^+^ T cells and tumor-CD8^+^ Treg cells as determined by flow cytometry. (**B**) Confocal images depicted nuclear translocation of GATA3 in control CD8^+^ T cells and tumor-CD8^+^ Treg cells (magnification 60x; scale bar: 5 μM) (*left panel*) and were analyzed statistically (*right panel*). (**C**) The relative changes in FOXP3 mRNA and protein were determined in GATA3-silenced control CD8^+^ T cells and tumor-CD8^+^ Treg cells before and after generation of CD8^+^ Treg in *in vitro* co-culture model. Transfection efficiency was determined by Western blot (*inset*). (**D**) rVista alignment of human/mouse/guinea-pig *FOXP3* genome. The conserved regions were depicted in red. The outlined area indicated GATA3-binding sites in CNS1 and 90% similarity for at least 35-bp length was considered for this analysis (*upper left*). In CNS2 region GATA3-binding site is 80% similar to 16 bp length sequence between human, mouse and guinea pig. CLUSTALW analysis depicted sequence conservation of GATA3-binding site on CNS1 (*bottom left*) and CNS2 (*bottom right*) enhancer region of *FOPX3* genome. (**E**) Schematic representation showed GATA3-binding site at CNS1 region (primer-1: +1392 bp to +1599 bp; *left panel*). The graphical representation showed relative binding of GATA3 in the *FOXP3*-CNS1 region in control CD8^+^ T cells and in tumor-CD8^+^ Treg cells generated at different time intervals (*right panel*). (**F**) Schematic representation of GATA3-binding site at CNS2 region (primer-2: +3381 bp to +3512 bp; *left panel*). Relative binding of GATA3 at the *FOXP3*-CNS2 region in control CD8^+^ T cells and in tumor-CD8^+^ Treg cells developed at different time intervals (*right panel*) were determined by ChIP assay. (**G**) (*Left panels*) Relative GATA3-binding and histone H4 acetylation at CNS1 region of GATA3-siRNA-transfected CD8^+^ T cells were determined by ChIP assay and ChIP-signals were compared with CD8^+^ Treg cells. (*Right panels*) GATA3 was silenced in CD8^+^ Treg cells and ChIP-signals for GATA3 and histone H4 acetylation was determined and was compared with CD8^+^ T cells. (**H**) GATA3-silenced CD8^+^ Treg cells were used to determine the ChIP-signals for GATA3 and histone H4 acetylation. Data are representative as the mean ± SEM and are the cumulative results of five independent experiments. *p < 0.05, **p < 0.01, ***p < 0.001.

Only transcriptional initiation is not sufficient to ensure the sustained function of a molecule; rather a stable expression is always required which also hold true for FOXP3 expression vis-à-vis the functional aspects of Treg cells. This information tempted us to look in-depth to the molecular mechanism associated with the maintenance of *FOXP3* expression in tumor-CD8^+^ Treg cells. GATA3 is an important transcription factor which plays an essential role in the maintenance of *Foxp3* expression in thymus-derived CD4^+^ nTreg cells^11^. GATA3 is over-expressed in Th2 cells and is regarded as the “master regulator” of Th2 phenotype^29^. Nevertheless, GATA3 function is not limited to Th2 cell only; it is constitutively expressed in CD8^+^ T cells also and helps in their functions^30, 31^. So, it was rational to assume that GATA3 might be playing some salient role in the regulation of *FOXP3* transcription in these cells. We checked it’s level and observed that GATA3 is significantly up-regulated in tumor-CD8^+^ Treg cells as compared to control CD8^+^ T cells (Fig. 5A). Our confocal microscopic data further showed its enhanced nuclear translocation (Fig. 5B) which gave an indication that it might be involved in the transcriptional regulation of *FOXP3*.

To understand the involvement of GATA3 in FOXP3 regulation more intricately, GATA3 was silenced in CD8^+^ T cells before the development of CD8^+^ Treg cells in *in vitro*-generated tumor model. Interestingly, it was observed that when GATA3 was ablated before CD8^+^ Treg generation, FOXP3 expression was enhanced significantly both at the transcription as well as translation levels (Fig. 5C). This data indicates that may be GATA3 is acting as a repressor for FOXP3 induction. But previous reports suggest that GATA3 is required to maintain expression of *Foxp3* in thymus-derived CD4^+^ Treg cells^11^. This information compelled us to study whether GATA3 is required at the later stages of CD8^+^ Treg development to continuously maintain the enhanced FOXP3 expression in these cells. For the same, we developed CD8^+^ Treg cells in *in vitro* tumor milieu and transfected them with GATA3-siRNA and cultured for further 48 h. Unlike the previous result, GATA3-ablation at a later stage, FOXP3 expression was reduced significantly in these cells both at transcriptional and translational level (Fig. 5C). Altogether these results indicate that GATA3 might be playing a dual role in the regulation of *FOXP3* transcription in tumor-CD8^+^ Treg cells.

The dual functions of GATA3 in the regulation of *FOXP3* in tumor-CD8^+^ Treg cells tempted us to understand the molecular mechanisms behind such bi-functional role of GATA3. To that end, we performed genome-wide analysis of GATA3-binding sites in a human *FOXP3* gene by rVISTA. Two putative GATA3-binding-sites were observed in CNS1 (+1448 bp) and CNS2 (+3488 bp) regions, and both the sites were found to be 80–90% conserved between human, mouse and guinea pig (Fig. 5D). As we observed a dual role of GATA3 in the regulation of *FOXP3* gene expression, we intend to unveil the relative binding of GATA3 in CNS1 and CNS2 region at various stages of CD8^+^ Treg development in our *in vitro* tumor model. It was evident from our ChIP assay, with primer set for CNS1 (Fig. 5E, *left panel*), that relative binding of GATA3 at CNS1 region was very high in control CD8^+^ T cells and gradually decreased with the development of CD8^+^FOXP3^+^ Treg cells (Fig. 5E, *right panel*). On the contrary, when GATA3-binding was assayed with primer set for CNS2 region (Fig. 5F, *left panel*), we observed a very low ChIP-signal in control CD8^+^ T cells and was significantly increased at 48 h (Fig. 5F, *right panel*).

To further support this idea, we investigated relative GATA3-binding and histone acetylation (Ac-H4) at CNS1 and CNS2 region in GATA3-siRNA-transfected cells. To this end, GATA3-silenced CD8^+^ T cells were used for ChIP assay at CNS1 region for GATA3 and Ac-H4. Results showed that in GATA3-ablated condition ChIP-signals for both GATA3 and Ac-H4 decreased significantly as compared to control-siRNA transfected CD8^+^ T cells (Fig. 5G, *left panels*). But when CD8^+^ Treg cells were RNA-interfered, the ChIP-signals for GATA3 and Ac-H4 at CNS1 region were not significantly altered (Fig. 5G, *right panels*). These data indicated that GATA3-binding and histone modification at CNS1 region are profound only in CD8^+^ T cells and might be responsible for the suppression of FOXP3 in naïve CD8^+^ T cells. On the contrary, in CNS2 region GATA3-ablation significantly inhibited ChIP-signal for both GATA3 and Ac-H4 in tumor-CD8^+^ Treg cells (Fig. 5H). All these results suggested a subtype-specific disparity in the regulation of CNS1 and CNS2 regions by transcription factor GATA3 in regulation of *FOXP3* expression.

**Figure 6:**
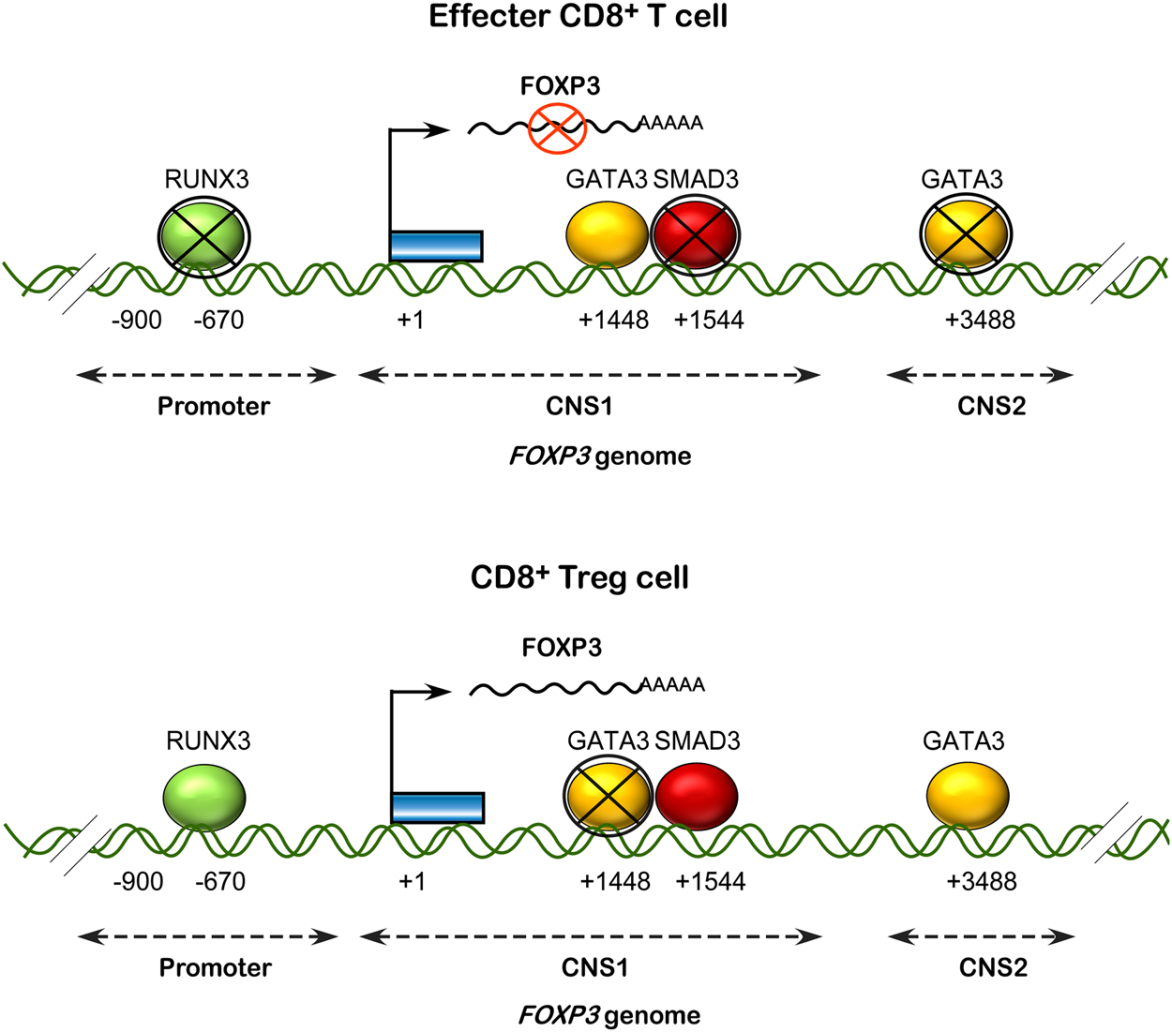
Transcription machinery of FOXP3 in CD8^+^ Treg cells. Schematic diagram showing the participation of RUNX3, SMAD3 and GATA3 in transcriptional regulation of *FOXP3*, in tumor-CD8^+^ Treg cells.

The cumulative results show the intricate transcriptional regulation of *FOXP3* is controlled by three transcription factors, SMAD3, GATA3, and RUNX3, involving CNSs and promoter regions of *FOXP3* in tumor-induced CD8^+^ Treg cells (Fig. 6).

## Discussion

In cancer immunotherapy, together with activating tumor-specific effector T cells, optimal strategies need to be established for attenuating Treg cells generation or their suppressive activity. Besides CD4^+^ Treg cell, CD8^+^ Treg cell is also a major player in developing tumor immunity^3, 4^. In the present study, we disclosed the mechanisms of development and function of CD8^+^ Treg cells in breast tumor microenvironment, emphasizing the transcriptional regulation of their *FOXP3* gene. Most of the previous studies have described the regulation of FOXP3 in CD4^+^ Treg cells; however, CD4^+^ and CD8^+^ cells have different lineage-specific transcription machinery which suggests that the regulation of FOXP3 in CD4^+^ and CD8^+^ Treg cells can differ. Therefore, research on the function and development of tumor-induced CD8^+^ Tregs is crucial, especially for their clinical application.

In tumor microenvironment, the CD8^+^CD25^+^FOXP3^+^ Treg population has found to be augmented with the advancement of breast tumor. FOXP3 being an intracellular molecule has limited value in the isolation of CD8^+^ Treg cells for its characterization. Various cell surface molecules were used to identify CD8^+^ Treg cells in different experimental systems, but no definitive signature molecules have yet been established. Here, we used FOXP3-transactivated cell surface protein, CTLA4, as a definitive phenotypic signature for tumor-CD8^+^ Treg cells. These CD8^+^ Treg cells, generated in the tumor microenvironment, produce high-level of immunosuppressive cytokines and inhibit effector T cell proliferation as well as their functions to ensure tumor immune evasion like conventional Treg cells^23^. Although the phenotypes of Treg cells are closely resembled with the activated or exhausted T cells, but it was observed that the CD8^+^ Treg cells generated in our *in vitro* tumor microenvironment were neither activated or exhausted CD8^+^ T cells. Interestingly, these CD8^+^ Treg cells act synergistically with CD4^+^ Treg in the suppression of effecter T cells which further emphasized the importance of the understanding of the development and function of tumor-induced CD8^+^ Treg cells.

We have witnessed a population of CD8^+^ Treg cells which express high-level of the transcription factor, FOXP3, in the tumor microenvironment. FOXP3 hijacks the transcription machinery for effector T cells and functionally converts them into regulatory cells but how the expression of this transcription factor is regulated in CD8^+^ Treg cells are not well documented yet. Thymocyte progenitors differentiate through a series of stages into mature T cells and RUNX3 binds to the *CD8* enhancer and contribute to transcription of *CD8* in CD8^+^CD4^−^ T cells during thymic selection and mature into CD8 T cell lineages^32^. Our genome-wide study by rVISTA multiple sequence alignment showed that *FOXP3-*promoter contains, in addition to other responsive elements, three conserved RUNX3-binding sites. Interestingly, it is to be noted that CD4^+^ iTreg cells also expresses a high level of RUNX which is responsible for the induction of Foxp3 in mice^12^. We also observed a similar phenomenon in CD8^+^ Treg cells those were generated in *in vitro* human breast tumor microenvironment.

There are three conserved noncoding sequences (CNSs) in the *FOXP3* locus, *viz*. CNS1, CNS2 and CNS3^26, 27, 33^, which act as enhancer regions and play an important role in the regulation of *FOXP3* transcription. Among them, CNS1 is critical for TGFβ-induced *FOXP3* transcription, whereas CNS2 is known to play a role in the maintenance of *FOXP3* expression in thymus-derived CD4^+^ nTreg cells^10, 28^. Since FOXP3 is precisely expressed in T-regulatory cells, it is obvious that its expression will be tightly regulated. So, apart from promoter activation, altered modulation of various enhancer regions in *FOXP3*-genome might be involved in controlling the induction and maintenance of FOXP3 in functional T-regulatory cells. In our previous studies, we observed that breast tumor cells produce an abundant amount of TGFβ which can induce *FOXP3* in the CD4^+^ naïve cell to differentiate them into Treg cells^2^. Likewise, our results also showed that FOXP3 induction in tumor-CD8^+^ Treg cells is TGFβ-dependent. Tone *et al*. observed that in the presence of TGFβ, SMAD3 binds to CNS1 region and induces *FOXP3* transcription in CD4^+^ T cells^34^. In line with this, our study has shown that in the tumor microenvironment, TGFβRI-induced SMAD3 activates CNS1 enhancer region of *FOXP3* genome with concomitant permissive^35^ chromatin modifications in CD8^+^ Treg cells. So, both the transcription factors RUNX3 and SMAD3 induced *FOXP3* by binding at the promoter as well as CNS1 regions in CD8^+^ Treg cells.

Only transcriptional initiation is not sufficient to ensure the optimal function of an FOXP3, rather a stable expression of this transcription factor is required for the immunosuppressive function of these Treg cells. Wang *et al*. had shown the role of GATA3 in the maintenance of stable *Foxp3* expression in thymus-derived CD4^+^ nTreg cells^11^, but no reports have yet been explored its role in inducible or iTreg cells. To our knowledge, we for the first time reporting that GATA3 plays a bi-functional role in the transcriptional regulation of FOXP3 in tumor-induced CD8^+^ Treg cells. In naïve CD8^+^ T cells, it binds predominantly at the CNS1 region and represses *FOXP3* transcription. This results can be explained by the fact that in type-2 cell polarizing condition GATA3 antagonizes TGFβ-induced *FOXP3* induction^36^, probably by repressing CNS1 enhancer activity. With the development of tumor-CD8^+^ Treg, GATA3 shows reduced affinity towards CNS1 region due to repressive chromatin modification and binds essentially to CNS2 region which displays permissive chromatin modification. These help in maintaining enhanced *FOXP3* expression in tumor-CD8^+^ Treg. Such bifunctional role of a transcription factor is not new. Reports suggest that depending on the cohorts of co-factors TBX20 transcription factor can act as an activator as well as a repressor of a distinct gene within adult heart^37^. Whereas transcription factor, NFY, can function both as a repressor and activator of von Willebrand factor expression through its different DNA-binding sequences^38^. Likewise, our study shows that GATA3 plays a dual role, both as repressor and activator in the transcriptional regulation of *FOXP3* depending upon its temporal binding on CNS1 and CNS2 regions.

In summary, our findings explore the generation and functions of tumor-induced CD8^+^ Treg cells that suppress the anti-tumor immunity alone or synergistically with CD4^+^ Treg cells indicating that targeting only CD4^+^ Treg is not sufficient to circumvent tumor immune evasion. Our results also suggest that the transcriptional regulation of *FOXP3* is controlled by three transcription factors, SMAD3, GATA3, and RUNX3, in a concerted manner involving CNSs and promoter regions of *FOXP3* in tumor-induced CD8^+^ Treg cells. This study suggests that cell-specific perturbation of transcriptional machinery can be used to restrain CD8^+^ Treg cell development and can potentiate effective anti-tumor immunity.

## Methods

### Cell isolation and cell culture

The present study included 20 female patients with breast cancer and 18–45y of age/sex/HLA-matched 12 female healthy volunteers. Informed consent (IRB-1382), under the provision of the human ethics committee, was obtained from all patients with localized disease and female healthy volunteers in compliance with Helsinki Declaration (http://www.wma.net/en/30publications/10policies/b3/). All experimental protocols were approved by Human Ethics Committee of SSKM Hospital (Approval No: Inst/IEC/306) and Bose Institute (Approval No: BIHEC/2014-15/6) and were carried out in accordance with the ethics committee. 60 percent of the collected tumor tissues were from triple-negative breast cancer and were of different stages starting from Stage-I to Stage-IV. We grouped Stage-I and Stage-II as early stage and Stage-III and Stage-IV as late stage. Out of 20 tumor samples, 17 samples showed positive responses, irrespective of the subtypes. Peripheral blood collected from healthy volunteers was centrifuged over lymphocyte separation medium (Histopaque, Sigma) to obtain total leucocytes. CD4^+^ and CD8^+^ T cells from healthy individuals were purified by magnetic selection using CD4^+^ and CD8^+^ magnetic beads (BD Biosciences, San Jose, CA). The purity of enriched cells was determined by flow cytometry and was consistent >95%. Cells were cultured in the complete RPMI-1640 medium at 37 °C in a humidified incubator containing 5% CO_2_.

To generate *in vitro* tumor microenvironment, tumor tissue was collected from breast cancer patients undergoing surgical procedures to remove solid tumor mass. The inner mass of tissues was cut into small pieces of 2–4 mm, digested at 37 °C for 3–4 h in a 1:1 solution of collagenase (Sigma-Aldrich, St. Louis, MO). After filtration through a 30-lm pore filter, single cells were plated in a single layer with RPMI-1640 medium supplemented with 10% fetal bovine serum for overnight. The purity of those tissue cells was checked flow cytometrically by CD24 and ESA-positivity or CD4/CD8 and CD25-negativity^2^. To mimic tumor microenvironment, adherent monolayer tissue cell bed was used for co-culture with CD8^+^ T cells that were isolated from a healthy donor. For the purification of tumor-CD8^+^ Treg cells, CD8^+^ T cells were first labeled with CTLA4-biotin antibodies (BD Bioscience, San Jose, CA) followed by addition of magnetic bead conjugated streptavidin particle and then subjected to magnetic separation. The purity of enriched cells was determined by CD8^+^CTLA4^+^ staining in flow cytometry and was consistent >95%.

### Cell treatment and transfection

To study the role of TGFβ signaling in tumour-induced CD8^+^ Treg cell development, isolated lymphocytes were treated with 100 nM SB431542 for 90 min, excess inhibitors were washed off, and cells were co-culture with tissue cells bed in the fresh RPMI-1640 medium. Cells were also treated with neutralizing anti-TGFβ antibodies (2 μg/ml; Santa Cruz Biotechnology, Dallas, TX). Control CD8^+^ cells or isolated tumor-CD8^+^ Treg cells were transfected with 300 pmol of SMAD3/RUNX3/GATA3/ FOXP3-siRNA/control-siRNA (Santa Cruz Biotechnology) and lipofectamine-2000^39^ (Invitrogen, Carlsbad, CA) followed by electroporation in single-pulsed method (voltage 260 V and capacitance 1050 μF) separately for 12 hr^1^. To check the transfection efficiency, respective protein levels were estimated by Western blot. The toxicity due to the lipofectamine-2000 mediated transfection was checked by Annexin-V/PI assay (Supplementary Fig. S3) and the live cell used for further experiments by using dead cell removal kit (Miltenyi Biotech, Singapore).

### Treatment of animals

BALB/c mice (NCLAS, Hyderabad, India) weighing 20–25 g were maintained in a temperature-controlled room with light-dark cycle. All animal experiments were performed in accordance with Principles of laboratory animal care (NIH publication No. 85–23, revised in 1985) as well as Indian laws on ‘Protection of Animals’. All experimental protocols were approved by Bose Institute Animal Ethics Committee for the purpose of control and supervision of experiments on animals (1796/GO/EReBiBt/S/14/CPCSEA, 18/12/2014). One group of mice were challenged with 1 × 10^6^ exponentially grown 4T1 cells in the mammary fat-pad. Tumor size was monitored regularly at 7 day interval. After 21 days, mice were sacrificed, and percentages of CD8^+^ Treg cells were determined in peripheral circulation, spleen and tumor-draining lymph nodes (popliteal).

### Flow cytometry

To study the phenotypes of CD8^+^ Treg cells, lymphocytes were incubated with the following antibodies: anti-CD8-FITC, anti-CD25-PE-Cy7, anti-CD127-AlexaFluor 647 and anti-CTLA4-PE (BD Biosciences). To analyze FOXP3 level, cells were fixed, permeabilized and labeled with anti-FOXP3-AlexaFluor-647 antibodies (BD Biosciences). The cells were analyzed by FACS Verse flow cytometer using FACS Suite software (BD Biosciences). To check phosphorylated-SMAD3, GATA3, RUNX3 level control CD8^+^/tumor-CD8^+^ Treg cells were incubated with pSMAD3, GATA3, RUNX3 primary antibody (Santa Cruz Biotechnology) followed by anti-mouse and anti-rabbit AlexaFluor 488 antibody and then level observed by flow cytometry (FACS verse). For the determination of intracellular cytokine, cells were first stimulated with PMA (10 ng/ml) and ionomycin (1 µM) and then treated with 10 µg/ml Brefeldin A (Sigma-Aldrich, St. Louis, MO). Intracellular TGFβ, IL10, IL2, and IFNγ were analyzed with the PE-tagged antibody (BD Biosciences) in FACS Verse.

### CFSE dilution assay

For T cell proliferation assay purified CD4^+^ T cells were loaded with CFSE (10 μM; Molecular Probes, Eugene, OR) and then co-cultured with *in vitro*-generated and patient-derived Treg cells at four different ratios, i.e, T-effector cell:Treg; 4:1, 2:1, 1:1, 1:2. The CD4^+^ T cell proliferation was also monitored in co-culture with a combination of both the CD4^+^/ CD8^+^ Treg subsets (1:1), in the presence of anti-CD3/CD28 beads. After three days responder T cells were subjected to CFSE dilution assay by flow cytometry^40^.

### Tc-mediated cytotoxicity study

To study the Tc mediated cytotoxicity, CD8^+^ T cells were first co-cultured with *in vitro-*generated and patient-derived CD8^+^ Tregs at Tc cell:Treg: 4:1, 2:1, 1:1, 1:2 ratios, then the Tc cells were purified by magnetic sorting (BD Bioscience) and further co-cultured with breast tumor cells (MCF7) for 48 h. Tc-induced tumor cell apoptosis was flow-cytometrically analyzed by Annexin-V/PI positivity.

### T cell apoptosis

To study the CD4^+^ effector-T cell apoptosis, the CD4^+^ T cells were isolated and co-cultured with *in vitro-*generated and patient-derived CD8^+^ Tregs at Tc cell:Treg:: 4:1, 2:1, 1:1, 1:2 ratios. After 72 h the effector T cells death was analyzed flow-cytometrically.

### Confocal microscopy

To examine the nuclear translocation of SMAD3 and GATA3, and the CTLA4 and FOXP3-positivity, cells were fixed and permeabilized. Then the cells were first incubated with anti-pSMAD3/GATA3 (rabbit-mAb), anti-CTLA4 (goat-mAb) and anti-FOXP3 (mouse-mAb) followed by fluorescence-tagged secondary antibodies (Invitrogen) and 4′,6-diamidino-2-phenylindole (DAPI; BD Pharmingen, CA). The cells were visualized in Leica confocal microscope, with DPX mounting medium, and at 60x magnification. Images were analyzed by ImageJ software^1^.

### RT-PCR and qPCR

Total cellular RNA was extracted using TRIzol reagent (Invitrogen Life Technologies, Carlsbad, CA), according to the manufacturer’s protocols (19). Amplification of 1 µg of the total RNA was conducted using gene-specific primers (Supplementary Fig. S2A). The reaction mixtures were then subjected to 45 cycles of denaturation at 95 °C for 15 s and annealing at 60 °C for 1 min. RTplus PCR system (Eppendorf, Hamburg, Germany) was conducted on a GeneAmpPCR 2720 (Applied Biosystems, CA, USA). Quantitative real-time PCR was performed in Master cycler gradient (an Applied Biosystems 7500 Sequence Detection System) using SYBR-green RoxMix (ABgene, Epsom, UK)^1^. Respective primers list was given in Supplementary Table: S1.

### Bioinformatics

Genomic sequences spanning the promoter and intronic region (UTR) of the Foxp3 gene were analyzed using the alignment software rVista 2.0: [http://rvista.dcode.org/] and CLUSTALW [www.genome.jp/tools/clustalw/], allowing to identify conserved regions as well as transcription factor binding sites, which uses matrices of the NCBI database (human accession NC_000023.11; mouse accession NC_000086.7; guinea pig accession NT_176287.1).

### Chromatin immunoprecipitation

Chromatin immunoprecipitation (ChIP) assays were carried out using a ChIP assay kit (Millipore, Billerica, MA) according to the manufacturer’s protocol. The specific antibody against SMAD3, GATA3, RUNX3 (Santa Cruz, Santa Cruz, CA) and Ac-H4 and Ac-H3 (Millipore, Billerica, MA) were used for respective ChIP assay. DNA was recovered from antibody–protein–DNA complexes by phenol/chloroform extraction and ethanol precipitation^1^. Then DNA fragments were amplified by PCR using respective primers (Supplementary Table: S1).

### Statistical analysis

Values are shown as the standard error of the mean (SEM) except when otherwise indicated. Comparison of multiple experimental groups was performed by 1-way ANOVA test followed by Bonferroni post-Hoc Test. Data were analyzed and, when appropriate, the significance of the differences between mean values was determined by Student’s t-test. Results were considered significant at P < 0.05.

## Electronic supplementary material

Supplementary information
