# Supplementary material for: Transcriptional regulation of FOXP3 requires integrated activation of both promoter and CNS regions in tumor-induced CD8+ Treg cells
Source: Sci Rep. 2017 May 9;7:1628. doi: 10.1038/s41598-017-01788-z (PMC5431671; doi:10.1038/s41598-017-01788-z)
Supplement: Supplementary file 1 — Supplementary information [file 41598_2017_1788_MOESM1_ESM.pdf]

Research article

Transcriptional regulation of FOXP3 requires integrated activation of both promoter and CNS regions in tumor-induced CD8$^+$ Treg cells

Sreeparna Chakraborty, Abir K. Panda, Sayantan Bose, Dia Roy, Kirti Kajal, Deblina Guha & Gaurisankar Sa*

[1]*Division of Molecular Medicine, Bose Institute, P-1/12, CIT Scheme VII M, Kolkata 700054, India*

Supplementary Information

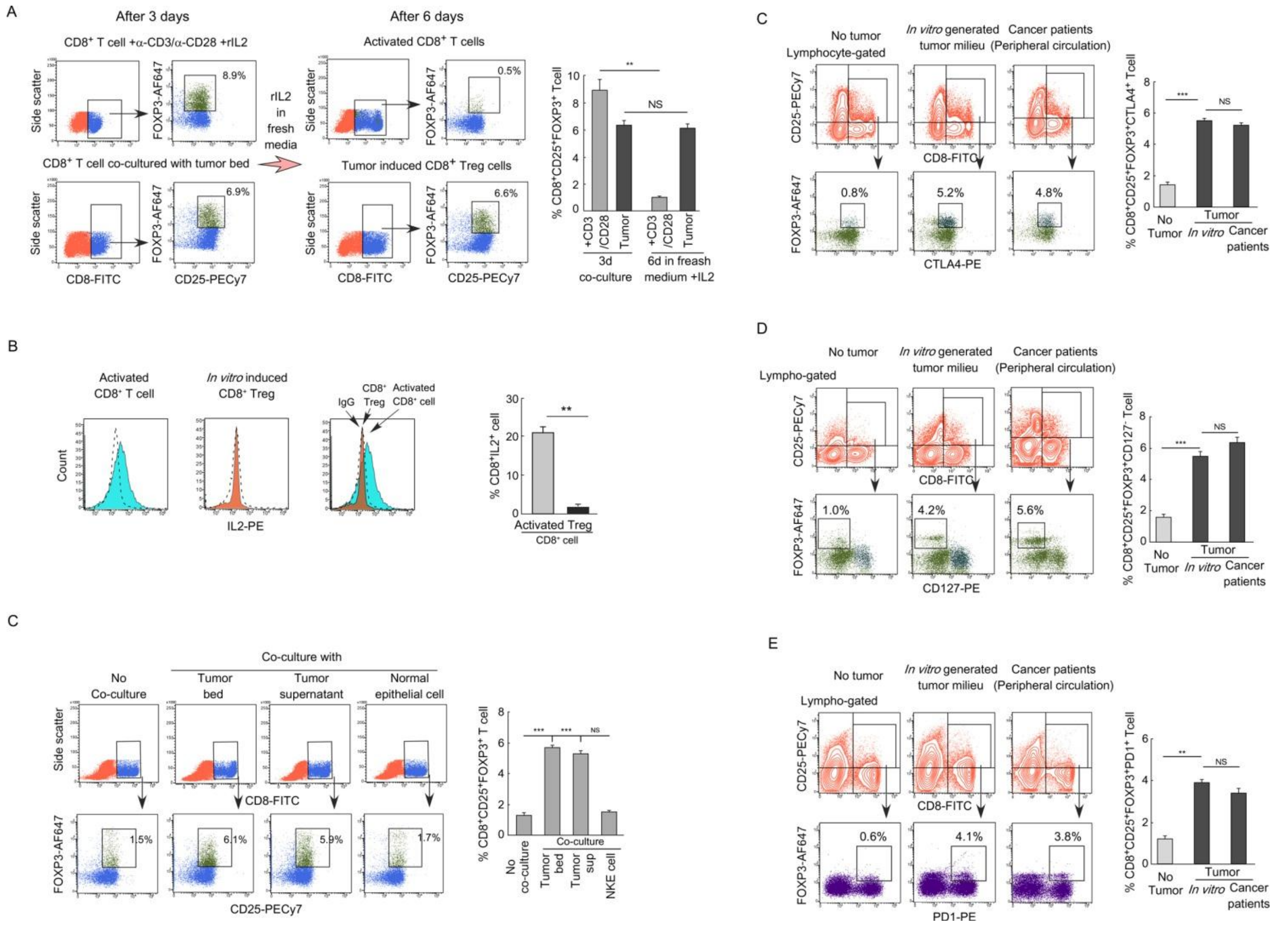

Supplementary Figure-S1: (**A**) The *upper left panel* depicted the induction of CD8$^+$CD25$^+$FOXP3$^+$ cells with CD3/CD28 stimulation and rIL2 treatment for 3 days and the *lower left panel* showed the percentage of the CD8$^+$CD25$^+$FOXP3$^+$ cells in *in vitro*-generated tumor microenvironment for 3 days. The *middle panel* depicted the percentage of those generated CD8$^+$CD25$^+$FOXP3$^+$ cells incubated in rIL2 supplemented fresh medium for 6 days. CD8$^+$ cells were gated to study the FOXP3-positivity in these cells and analyzed statistically (*right panel*). (**B**) The IL2-positivity was assayed within activated CD8$^+$ T cells and in *in vitro*-generated CD8$^+$ Treg cells and presented graphically. The dotted line showed the isotype control. (**C**) The flow cytometry data showed the percentage of CD8$^+$ Treg cell within total lymphocyte population present in a healthy individual, and generated in *in vitro* co-culture with tumor tissue bed, with tumor tissue supernatant and with normal epithelial (NKE) cells for 72h (*left*). The bar diagram represented the percentage statistically (*right*). The percentage of (**D**) CD8$^+$CD25$^+$FOXP3$^+$CTLA4$^+$ cells, (**E**) CD8$^+$CD25$^+$FOXP3$^+$CD127$^-$cells, and (**F**) CD8$^+$CD25$^+$FOXP3$^+$PD1$^+$ cells present in the peripheral circulation of a healthy donor, cancer patients and in *in vitro*-generated tumor microenvironment were determined by flow cytometry and analyzed statistically. Within the lymphocytes, the CD8$^+$CD25$^+$ population was gated to study FOXP3$^+$CTLA4$^+$/FOXP3$^+$CD127$^-$/FOXP3$^+$PD1$^+$ cells within it and the percentage showed within total lymphocytes. Isotype-matched control antibodies were used for all flow cytometric experiments. Data are representative as the mean ±SEM and are the cumulative results of five independent experiments. **p<0.01, ***p< 0.001, NS: non-significant.

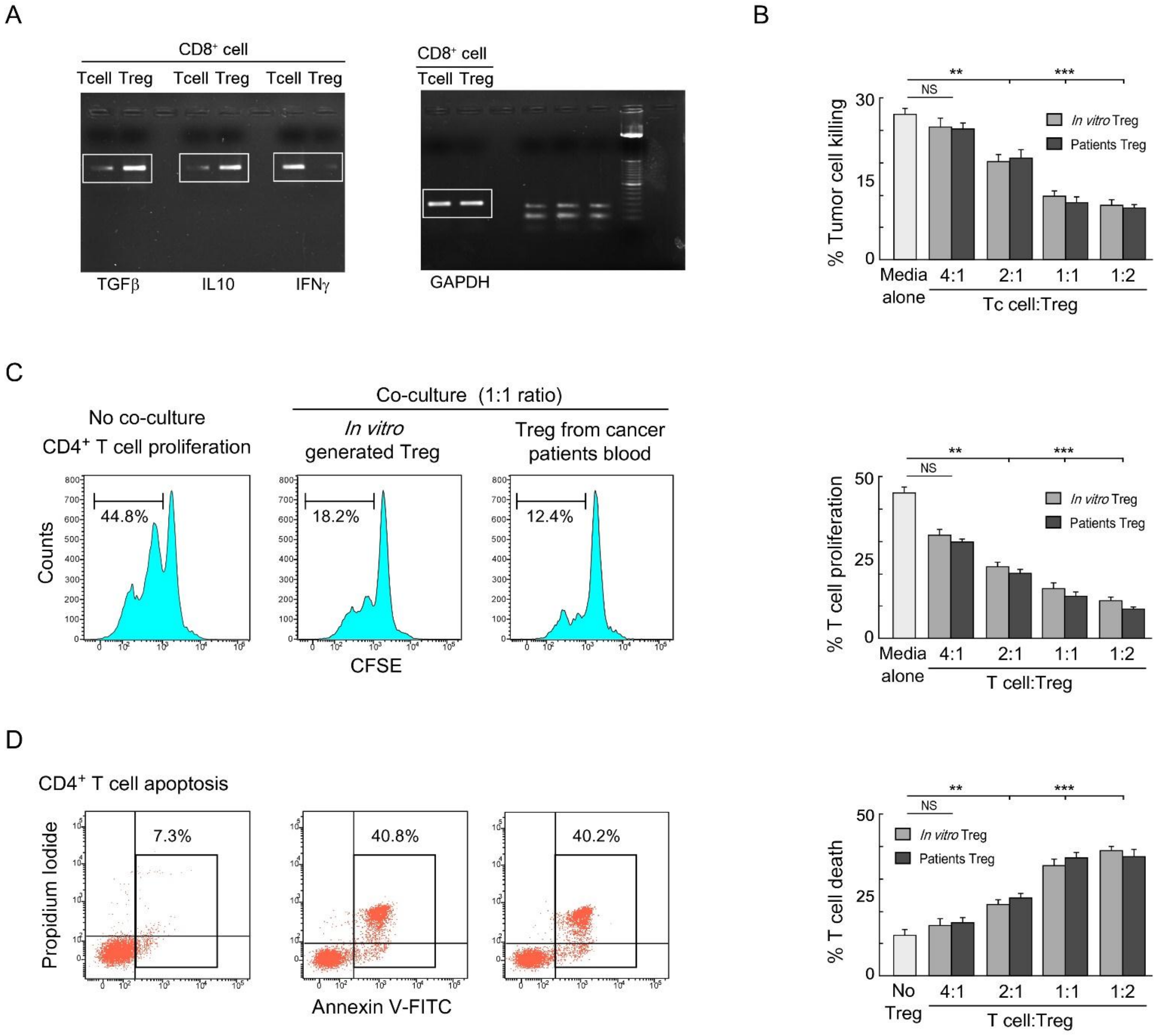

Supplementary Figure-S2: (**A**) The RT-PCR data shows the cDNA level of TGFβ, IL10, IFNγ (*left panel*) and GAPDH (*right panel*) transcripts in control CD8+ T cells and CD8+ Treg cells. The box indicates the cropped portions that were used in Figure-2 of main article file. (**B**) CD8+ cytotoxic cells (Tc) were primed with in vitro generated and patients' CD8+ Treg cells. Control and Treg-primed Tcs were then co-incubated separately with breast tumor cells for 48h and apoptotic index of the tumor cells i.e. CD8-negative population were analyzed flow cytometrically by Annexin-V/PI-positivity. Suppressive activity of Treg cells was measured in co-culture with Tc cells at 4:1, 2:1, 1:1 and 1:2 ratio (Tc cell: Treg) and represented statistically. (**C**) CD4+ responder T cell proliferation was measured by CFSE-dilution assay in presence and absence of isolated CD8+ Treg cells generated *in vitro* tumor microenvironment or in the peripheral circulation of cancer patients (*upper left panel*). The suppression was measured in co-culture at a 4:1, 2:1, 1:1 and 1:2 ratio (T effecter cell: Treg) *(upper right panel)*. (**D**) The CD4+ T cells death in presence and absence of *in vitro*-generated CD8+ Treg cells or CD8+ Treg cells isolated from cancer patients were analyzed flow cytometrically by Annexin-V/PI-positivity *(lower left panel)*. The suppression was measured in co-culture at a 4:1, 2:1, 1:1 and 1:2 ratio (T effecter cell: Treg) and represented statistically *(lower right panel)*. Isotype-matched control antibodies were used for all flow cytometric experiments. Data are representative as the mean ±SEM and are the cumulative results of five independent experiments. **$p<0.01$, NS: non-significant.

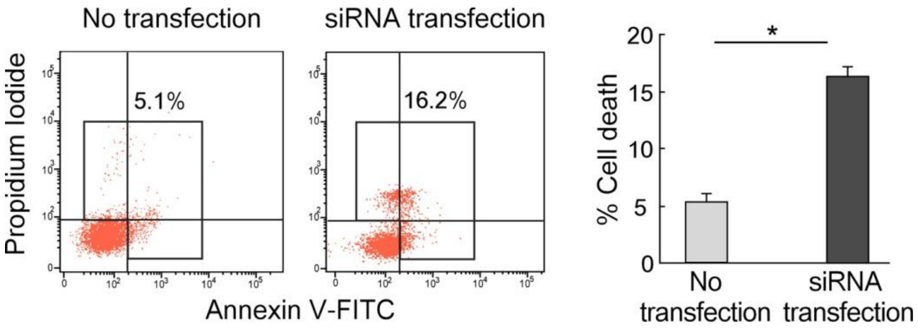

Supplementary Figure-S3: The flow-cytometry dot plot represented the Annexin-V/propidium iodide positive cells in non-transfected and siRNA-transfected CD8+ cells (*left*) and showed statistically in *right*. Data are representative as the mean ±SEM and are the cumulative results of five independent experiments. *p<0.05.

Supplementary Table-S1. Primers used for different experiments

	**Forward Primer**	**Reverse Primer**
Gene	**Primer for RT-PCR and qPCR**	
IL10	5'-GCTTGGGGCTTCCTAACTG-3'	5'-GTTGGGGAATGAGGTTAGGG-3'
TGFβ	5'-AGCCTGAGGCCGACTACTAC-3'	5'-CGTGCTGCTCCACTTTTAAC-3'
FOXP3	5'-CAAATGGTGTCTGCAAGT-3'	5'-CACAGATGAAGCCTTGGT-3
GAPDH	5'-AAGGTGAAGGTCGGAGTCAA-3'	5'-ATGACAAGCTTCCCGTTCTC-3'
FOXP3-genome	**Primer for ChIP assay**	
Promoter (RUNX-720)	5'-GGTTGGCCCTGTGATTTATT-3'	5'-ATTTCTTCCCCTCACCACA-3'
Promoter (RUNX3-670)	5'-TATCAGCGCACACACTCATC-3'	5'-CTGGCTTGTGGGAAACTGT-3'
Promoter (RUNX3 -441)	5'-GTGGTGAGGGGAAGAAATCATA-3'	5'-GATGAGTGTGTGCGCTGATAA-3'
SMAD3 (CNS1 site)	5'-AGGTTAAGAGTGTGGGTACTGG-3'	5'-TGAGGAAATGGAGGTATGGA-3'
GATA3 (CNS1 site)	5'-TGGCTTCTGTCTCTGGTTTT-3'	5'-GGCTCCAGTACCCACACTC-3'
GATA3 (CNS2 site)	5'-GGACATCACCTACCACATCC-3'	5'-ACCACGGAGGAAGAGAAGAG-3'
